# The netball injury evidence base: a scoping review of methodologies and recommendations for future approaches

**DOI:** 10.1186/s13643-024-02629-7

**Published:** 2024-08-01

**Authors:** Sara Horne, Aliah Faisal Shaheen, Bill Baltzopoulos, Laura Hills

**Affiliations:** 1 Department of Life Sciences, Division of Sport, Health and Exercise Sciences, Brunel University London, Kingston Lane, Uxbridge, Middlesex UB8 3PH UK; 2 School of Sport and Exercise Sciences, Faculty of Science, Liverpool John Moores University, Canterbury, UK

**Keywords:** Netball, Injuries, Scoping Review, Epidemiology, Sport, Incidence, Risk Factors, Mechanisms

## Abstract

**Background:**

Netball is a sport with a large participation base and a high risk of injuries. Effective injury prevention strategies are dependent upon a clear understanding of injury issues, aetiology and mechanisms, requiring robust research methodologies to ensure a reliable evidence base. This scoping review aims to identify the characteristics and range of netball injury research methodologies, to inform recommendations for future research.

**Methods:**

A systematic search of SPORTDiscus, MEDLINE, CINAHL and Academic Search Complete, PubMed, Scopus and Web of Science, from 1985 to May 2023 identified relevant studies. Inclusion criteria included peer-reviewed studies assessing injury incidence, aetiology and mechanisms in netball.

**Results:**

Following screening, 65 studies were included (68% descriptive epidemiology, 32% analytic epidemiology). Descriptive epidemiology reported data from hospital/clinic and insurance databases (57%) and netball competitions (43%). Only two studies used ongoing, systematic injury surveillance in netball cohorts, and significant heterogeneity existed in study designs, data collection methods, injury definitions and injury incidence rates calculations. Studies assessed a limited number of risk factors (descriptive competition studies: median: *n* = 4; analytic studies median: *n* = 6), with 76% using a simplistic reductionist approach to determine causality. Basic descriptions and retrospective recall of injury mechanisms reduced accuracy. Only two studies conducted comprehensive assessments of injury mechanisms using video-based methods.

**Conclusion:**

To establish an accurate netball injury evidence base, future research should prioritise the development of reliable, continuous surveillance systems. The International Olympic Committee (IOC) consensus statement guidelines are recommended for accurate injury data collection and reporting. A multifactorial approach should be adopted to assess the complex interaction between multiple risk factors, player load and the injury inciting event. Comprehensive descriptions of injury mechanisms using video methods, alongside descriptions from medical staff are recommended. This information is crucial for developing targeted prevention strategies.

**Supplementary Information:**

The online version contains supplementary material available at 10.1186/s13643-024-02629-7.

## Background

Netball is a popular court-based team sport, played predominantly by females. The international governing body reports over 20 million participants across 117 nations spanning Africa, Americas, Asia, Europe and Oceania, with ongoing global growth [1]. However, netball’s intermittent, dynamic nature, involving repeated high-intensity sprints, jumps, landings, cuts and changes of direction [2–5], imposes considerable physical demands on players. These actions, combined with netball’s unique footwork rule, generate substantial forces [6–8] and player workloads [9–13]. Consequently, injury rates are high, ranging from 11.3–14 injuries/1000 player hours (h) at the community level [14–17], to elite rates from 54.8/1000 h at the 2019 Netball World Cup [18] up to 500.7/1000 h [19] in South African players. Hence, effective prevention strategies are crucial to support growing participation and minimise the negative impact of injuries at all levels.

Sports injury research, guided by van Mechelen et al.’s. ‘sequence of prevention’ [20] and the Translating Research into Injury Prevention Practice (TRIPP) [21] models, emphasises the importance of identifying the injury evidence base to inform prevention strategies. Hence, the initial crucial steps involve understanding the sport's injury problem through injury surveillance [22], followed by identifying the risk factors and mechanisms causing injuries [23, 24]. To ensure prevention strategies are effective, it is essential to collect accurate evidence using robust data collection methods. This requires the continual, systematic collection of high-quality data from injury surveillance systems across various settings [22], and a multifactorial approach to understand the complex interactions between multiple risk factors and injury mechanisms [23, 24].

Currently, there is limited review evidence describing the characteristics of methodologies used in netball injury research. Two recent netball reviews provide valuable synthesis of injury types, characteristics and risk factors, but only briefly address methodological considerations [25, 26]. Therefore, there is an urgent need for a comprehensive review of the methodologies used in netball injury research to establish the injury evidence base. Furthermore, while the recent consensus on netball video analysis framework [27] provides guidance for the assessment of injury mechanisms from match video, there is currently no consensus statement to inform injury surveillance methods in netball. Consequently, a scoping review of this area was considered appropriate to provide researchers with an overview of existing netball injury methodologies and to inform future research directions.

Therefore, the purpose of this scoping review is to evaluate the range and characteristics of methodologies used to describe 1) the incidence, severity and burden of injuries 2) the aetiology and mechanisms of injuries in netball. This information will be used to provide recommendations for future research to ensure the accuracy of the evidence base for targeted netball injury prevention.

## Methods

### Protocol

This review was conducted in accordance with the Preferred Reporting Items for Systematic reviews and Meta-analyses extension for Scoping Reviews (PRISMA-ScR) and PRISMA 2020 updated statement [28, 29] (see Additional file 1 for PRISMA-ScR checklist).

#### Data sources and search strategy

A systematic, structured search strategy was developed with the assistance of a subject-specialist librarian. The electronic databases searched were SPORTDiscus, MEDLINE, CINAHL and Academic Search Complete (EBSCOhost), PubMed, Scopus and Web of Science from 1985 to 24th May 2024. The start date of 1985 was selected as Hopper (1986) [30] is recognised as the first peer-reviewed study on netball injuries [25]. The search terms used in all databases were “Netball*” AND “Injur*” AND (“incidence” OR “prevalence” OR “epidemiolog*” OR “risk*” OR “mechanism*” OR “cause*”). A secondary search of reference lists of included papers and Google Scholar was conducted to locate any additional studies eligible for inclusion.

#### Study selection

Following the removal of duplicates, the titles and abstracts were independently screened by two authors (SH, AFS) using the eligibility criteria. All articles that could not be excluded from this process were retrieved and underwent full-text screening. Where disagreements occurred, both authors met and discussed the studies until a consensus was gained. Hence, a third author was not required.

#### Eligibility Criteria

Eligible studies included those reporting data on netball injuries across all ages and levels of competition. These studies investigated the incidence, severity and burden, and/or the aetiology (risk factors) and mechanisms of netball injuries. Only studies published in English and peer-reviewed journals were included. Studies were excluded if they did not investigate netball, or they assessed the efficacy of prevention strategies, biomechanical factors in netball players un-related to injuries, or the physiological/movement demands of the game. Analytical studies that included netball athletes as part of a broader sports cohort but generalised findings across sports were also excluded e.g. Rigg et al. [31] and Almousa et al. [32]. Additionally, review articles, consensus statements, abstracts, and reports were excluded. All definitions of netball injuries were accepted. As outlined in the injury prevention literature [22, 24], aetiology is defined as the causes or risk factors that lead to injury. The injury mechanism is defined as the inciting event (playing situation and athlete behaviour) and biomechanical features resulting in injury [22].

#### Data extraction and analysis

Authors (SH, AFS) reviewed the included studies and discussed their categorisation, which was subsequently agreed by all authors. Studies were classified as descriptive epidemiological (describing the incidence and nature of netball injuries) or analytic epidemiological studies (identifying the association between specific risk factors and netball injuries or injury mechanisms), in a similar approach to Pluim et al. [33]. The descriptive epidemiological studies were further classified according to study design as studies using hospital/clinic records and insurance claim databases (hospital/clinic and insurance studies), or studies using injury data from netball competitions and/or historical injury data of match-play (netball competition studies). All studies were also classified by study design as prospective cohort, retrospective cohort or cross-sectional studies.

Data extraction from the included studies was conducted by the main author. Subsequently, the data from 14 studies (22%); descriptive epidemiology *n* = 10 (23%); analytic epidemiology *n* = 4 (19%), were verified by a second author (AFS). The data extracted included study details (author(s) and publication date), study design and data collection methods, data collection period, country of origin, population (including level, age and sample size), injury definitions and classifications, injury incidence and exposure, body regions, risk factors assessed and data analysis methods. Only those risk factors specifically related to netball injury data were included. The findings are summarised quantitatively with frequencies and percentages mapping the extent, nature, geographical distribution and range of methodologies in the studies.

## Results

### Study Selection

**Fig.1:**
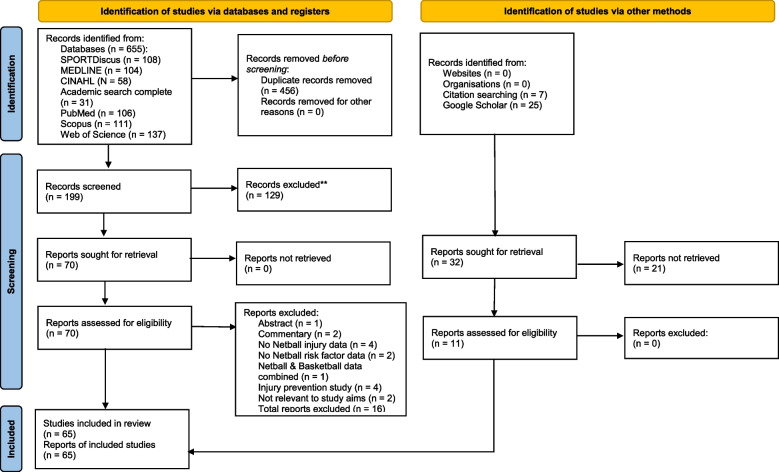
Flowchart of scoping review selection process

The database search yielded 655 studies, reduced to 199 following the removal of duplicates. After screening the titles and abstracts, 70 studies were identified for full-text screening. A further seven studies were identified through a secondary search of reference lists and 25 from Google Scholar, with 11 selected for full-text screening. Thus, a total of 81 studies received full-text screening. Subsequently, 65 studies were identified for inclusion in the review. A flowchart of the study selection process is shown in Fig. 1.

### Review Findings

Tables 1, 2 and 3 provide a summary of the findings based on the study categories. Each table describes the study design and data collection methods, data collection period, country of origin, population, injury definitions, injury incidence and exposure methods and body region. The findings are also presented in graphical and tabular formats in Additional File 2.

**Table 1:** Methodological details and injury incidence of netball descriptive epidemiological studies using hospital records, clinic records and insurance claim databases

Study	Country	Study design & data collection methods	Data collection period	Population	Injury definitions	Injury proportion or rate	Body region
Purdam (1987) [34]	AUS	Prospective study: Data from physiotherapy dept at AIS	1986 10 m	Elite Netball and Basketball players at AIS. 20 Netball players, 105 inj	All injuries presented to physiotherapy dept for treatment. Severity: based on no. treatments. Recurrent inj: inj > 1 m from discharge	Rate: 5.25 inj/player/y	All
Hume (1993) [35]	NZ	Retrospective study: Data from HSS hosp morbidity data, Dunedin hospital A&E, ACC claims & Dunedin Sports injury clinic	1988–1992 1 y Clinic 1.7 y	Total population of Netball players in New Zealand, estimated to be 98, 680 in 1989–1990 age: 15 + y and children 5–15 y, 143 netball hospitalisations, age 5 + y	Any inj reported to hospital ED, ACC, sports injury clinic or recorded in health statistics. Severity: Abbreviated Injury Severity score, minor, moderate or severe	Netball hosp rate: 4.3/100,000 population/y 143/100,00 persons/y	All
Hume & Marshall (1994) [36]	NZ	Retrospective study: Data from HIS mortality data, Dunedin hospital A&E, ACC & Dunedin Sports injury clinic claims	1978–1990 1 y Mortality 10 y	Netball population: 155,592 netball participants, 139 hospitalisations, age 15 y +	All injuries occurring in a place of recreation & sport, involving organised sporting activity or training for such presenting to hospital ED, ACC, sports injury clinic’ Severity: Abbreviated Injury Severity score, minor, moderate or severe	Netball hosp rate: 89.34/ 100,000 population	All
Finch et al. (1998) [37]	AUS	Retrospective study: Hospital ED data collated by NISU. Standardised form self-report, inj diagnosis and treatment completed by doctor	1989–1993 5 y	General population: 98,040 sports and active recreation participants presenting to ED’s. 2165 child (< 15 y), 3098 adults (≥ 15 y) netball presentations	All injuries presented to hospital ED. Severity: proxy measure, hosp admission after ED attendance	Proportion: Child netball inj 3.7%; adult netball inj 6.6% of all sport inj presentations	All
Love et al.(1998) [38]	NZ	Retrospective study: Data obtained from NZ ACC database of sport injury dental claims	1993–1996 4 y	General population: 260 netball claims. Age 0–75 +	Any new and minor dental claims made during each year. New claims are those that paid the claimant. Minor claims paid the health professional but not the claimant	Rate: 260 netball dental claims/y	Dental
Hassan & Dorani (2001) [39]	UK	Prospective study: All sports-related fractures reported to A&E Dept in a district hosp in NE, ENG	1997–1998 1 y	General population: 1255 children with sport inj, 54 netball presentations Age 5–15 y	Any fracture while participating in sport that led to presentation at A&E. Severity: Injury Severity Score (ISS)	Proportion: 24% of A&E netball inj were fractures	All
Hon et al. (2001) [40]	MAL	Prospective study: All sports-related fractures presenting at Dept of Orthopaedic & Traumatology in a state hosp in W, MAL	1998–1999 1 y	General population: 113 patients presenting with fractures, 3 netball players. Age 7–59 y	All fractures sustained during sports activity presenting to Dept of Orthopaedic & Traumatology	Proportion: 2.7% of fractures in netball players	All
Cassell et al. (2003) [41]	AUS	Retrospective study: Data from hosp admissions (VAED dataset), hosp ED presentations (VISS) & GP presentations (ELVIS project) in area of a regional hosp in Victoria, AUS	1994–1995 1 y	General population: estimated 69,663 in study area. 2300 medically treated sports inj; netball 81ED, 67 GP Age 4 + y	All sporting injuries receiving medical treatment recorded by 3 injury surveillance systems. Severity: length of hosp stay Injury Classification: ICD	Proportion: ED 6.9%; GP 6.7% netball inj presentations	All
Chong & Tan (2004) [42]	SNG	Retrospective study: Data from medical records & telephone interviews of all ACL reconstructions from Dept Orthopaedic Surgery in E, SING	1999–2002 4 y	General population: 259 patients with ACL ligament reconstruction: 13 female patients, 4 netball players (3 school, 1 club). Age 13–38 y	All female ACL injuries requiring reconstruction	Proportion: 31% ACL inj to netball players	Knee
Otago & Peake (2007) [43]	AUS	Retrospective study: Accepted netball insurance claims in Victoria, AUS	1999 1 y	Total population of registered state netball players covered by insurance scheme: total 87,331. 829 insurance claims. Age 10 y +	All inj resulting in accepted insurance claims Severity: cost of injury	Rate: 9.49 inj/1000 netball players	All
Smartt & Chalmers (2009) [44]	NZ	Retrospective study: Data from Netball inj hosp records, linked public hospital discharges, and ACC claim datasets	2000–2005 6 y	Estimated population of netball participants in New Zealand: 200,000 players, 1126 netball inj cases. Age 5 y +	All netball inj cases recorded in hospital discharge datasets. Severity: Injury Severity score (ICISS) Injury Classification: ICD	Rate: 5/100,000 netball participants	All
Flood & Harrison (2009) [45]	AUS	Retrospective study: Netball and basketball inj resulting in hosp admission, data from National Hospital Morbidity Database	2000–2004 4 y	Netball and Basketball population estimates. 5090 basketball-related, 4596 netball-related hospital admission. Age 5–54, mean 26.3 ± 10.9 y	All netball and basketball patients discharged from a private or public hospital Injury Classification: ICD	Av annual hosp rates: Netball 1.4/1000 participants	All
Gianotti et al. (2009) [46]	NZ	Retrospective populationbased study: Knee ligament inj data from ACC records	2000–2005 5 y	General population of New Zealand: approximately 4.1 million people. 3997 sport-related inj: 746 netball. Age 0–85 y +	Any personal knee ligament injury resulting in an ACC claim made at time of medical treatment	Proportion: 18.7% of all sport-related ACLS inj	Knee
Welch et al. (2010) [47]	NZ	Retrospective case series: Data on sports-related dental injuries from ACC records	1999–2008 10 y	General population of New Zealand: Active adults 2.7 million, 700,000 young people. 275,130 new claims. Age range 0 – 61 y +	All new oralfacial ACC claims received in financial years 1999 to 2008	Proportion: netball 3.9% of all sport-related claims	Dental
Gwynne-Jones et al. (2011) [48]	NZ	Retrospective study: Data from ED, in-patient, surgical audit and physiotherapy dept records	1999–2008 8.5 y	General population: 363 patients. 285 sport-related inj, 88 netball players. Age 15–60 y	A complete, traumatic closed rupture of the Achilles tendon in hospital patients. Recurrent inj: re-ruptures	Proportion: netball 24% of inj	Achilles Tendon
Jansen et al. (2011) [49]	AUS	Retrospective study: ACLR data from National Hospital Morbidity Database	2003–2008 5 y	General population: 50,187 patients with ACLR. Annual netball ACLR 1085. Age 5–75 y +	All ACLR in study period concerning the population Injury Classification: ICD	Annual ACL reconstruction rate: 188/100,000 participants	Knee
Fernando et al. (2018) [50]	AUS	Retrospective case series: Sport & recreation injuries presented to ED’s across 38 hosp in Victoria, AUS, data recorded in Victorian Emergency Minimum Dataset	2012–2015 3 y	General population: 171,541 ED presentations, 5438 Netball. Age 5 y +	All sports & recreation injuries reported to ED’s	Annual inj rate: 38.7/100,000 participants	All
Joseph et al. (2019) [51]	AUS	Retrospective study: Netball specific inj recorded in national insurance claim database over 1 season	2016 1 y	All players registered to play in Netball Australia organised competition receiving insurance cover. Total participants 413,800 players. 1215 netball inj claims. Mean age 34 ± 17 y	Any netball injury resulting from an accident during matches or training for an organized Netball Australia competition Injury Classification: OSIICS	Annual inj rate: 2.9 claims per 1000 participants	All
King et al. (2019) [52]	NZ	Retrospective study: Sport-related inj data from ACC. Inj claims from 5 sports, including netball	2012–2016 5 y	General population: 853,824 total claims. 11,757 total netball claims	Any injury assessed by a registered health practitioner as a result of sports participation. Severity: cost of injury	11,748 moderate-toserious inj claims; 9 serious inj claims	All
Kirkwood et al. (2019) [53]	UK	Retrospective ecological study: Sports injury data from ED data & in-patient data from 2 hosp in Oxfordshire, ENG	2012–2014 2 y + 2 m	General population: Children and adolescents attending hosp; 11,676 sports inj ED attendances. Age 0–19 y	Any sports-related inj attendances at AE depts. Injury Classification: ICD	157 netball injuries	All
Sutherland et al. (2019) [54]	NZ	Cross-sectional study: Sports injury ACLR data from ACC	2009–2016 8 y	General population: 20,751 male and female ACLRs. Netball 3088 claims. Mean age 29 ± 11 y	Any injuries involving claims made for primary ACLR	Proportion: netball 20% of sports-related ACLR claims	Knee
Belcher et al. (2020) [55]	NZ	Retrospective study: Audit of netball injury ACC claims	2008–2017 10 y	Total population of Netball New Zealand affiliated members. Age 10–24 y	New netball-related claims involving 4 treatments or more, or cost > $100NZD	Rate (10 y): Ankle 77.8/1000 players, Knee 71.6/1000 players	Ankle & Knee
Chan et al. (2021) [56]	SING	Retrospective study: ACLR data from electronic medical records & registry data of tertiary public hospital in SING	2013–2016 3 y + 6 m	General Asian population: 696 male and female patients, 21 netball inj Mean age 25.7 ± 7.2 y	All ACL injuries involving primary ACLR, on ACLR registry	Proportion: netball injuries 4.3% of all ACLR patients	Knee
Mitchell et al. (2021) [57]	UK	Cross-sectional study: Acute sports-related inj presented to fracture clinic at Peterborough city hospital, ENG	2018–2019 1 y	General population of school age children, 54 netball inj. Age 6–18 y	All sports-related inj in school age children, reporting to fracture clinic. Severity: surgical treatment equals severe injury	Proportion: 2% required surgery, 11% required physiotherapy	All
Brimm et al. (2023) [58]	AUS	Retrospective study: Sports-related hosp in Queensland, AUS	2012 – 2016 5 y	General population: 76,982 hosp. Netball 1150 hosp. Age: children ≤ 14 y to older adults ≥ 65 y	Any patients with sports & leisure-related inj admitted to public and private hospitals. Injury Classification: ICD	Rate: Total 4.9/100,000 Females: 8.8/100,000 Males: 111, 0.9/100,000	All

*AUS* Australia, *NZ* New Zealand, *UK* United Kingdom, *ENG* England, *MAL* Malaysia, *SING* Singapore, *AIS* Australian Institute of Sport, Inj, injury/injuries, *Hosp* Hospitalisation, *y* years, *m* month, *SD* standard deviation, *HSS* Health Statistical Services, *ACC* Accident Compensation Corporation, *A&E Dept* Accident and Emergency Department, NE; *HIS*, Health Information Service, *NISU* Australian National Injury Surveillance Unit;, North East, *W* Western, *VAED* Victorian Admitted Episodes Dataset, *ED* Emergency Department, *VISS* Victorian Injury Surveillance System, *GP* General Practitioner, *ELVIS* Extended Latrobe Valley Injury Surveillance, *ICD* International Classification of Diseases, *E* Eastern, *ACLS* Anterior Cruciate Ligament surgeries, *ACLR* Anterior Cruciate Ligament reconstruction, *IRR*, Incidence Rate Ratio’s

### Study Design

Of the 65 studies included in the review, 44 (68%) were descriptive epidemiological studies, while 21 (32%) were analytic epidemiological studies. The descriptive studies utilised injury data from hospital/clinic records and insurance databases in 25 studies (57%) (Table 1), while 19 studies (43%) collected data from netball competitions (Table 2). Most descriptive studies assessing hospital/clinic records and insurance data were retrospective in design (*n* = 20, 80%), while the netball competition studies more frequently utilised prospective study designs (*n* = 12, 63%) Similarly, most analytic studies (Table 3), were prospective in design (*n* = 11, 52%) with 7 (33%) using cross-sectional designs (Additional File, 2 Fig. 1). One analytic study reported both retrospective and prospective injury data [73], hence a total of 22 analytic epidemiology study designs are reported.

**Table 2:** Methodological details and injury incidence of netball descriptive epidemiological studies of competitions

Study	Country	Study design & data collection methods	Data collection period	Population	Injury definitions	Injury proportion or rate & athlete exposure	Body region
Hopper (1986) [30]	AUS	Prospective cohort study: State competition: Junior 124, Senior 324 teams. Questionnaire: Self report + physio post treatment	1983 1 × 14 wk season	Recreational to competitive level. 3,108 netball players, 158 inj. Age Junior: 12–15 y; Senior 16 y +	Any injury presenting to First Aid room requiring immediate medical care or with some form of disability. Minor inj not included	Rate: 50.82/1000 players/match	All
Hopper et al. (1995a) [59]	AUS	Prospective cohort study: State competition Questionnaire: Self report + physio post treatment	1985–1989 5 × 14 wk seasons	Recreational to competitive level. 11,228 netball players, 608 inj. Age 14 y + mean 18.8 ± 5.6 y	Any inj presenting to First Aid room requiring immediate medical care or presented with some form of disability. Minor inj not included. Severity identified by Physio, graded 1, 2 or 3 based on symptoms	Rate: 0.054/player/match	All
McKay et al. (1996) [60]	AUS	Prospective cohort study: Netball and basketball State competitions. Trained observers recorded inj, players completed questionnaire & follow-up telephone interviews	1991–1992 2 × seasons	Elite & recreational level. 9,190 netball players, 159 inj. Mean age 27.2 ± 7.8 y	Bodily harm to player causing stoppage of play, substitution or obvious disability. Severity classified on time-loss and treatment graded trivial, minor, substantial, major, severe	Rate: 17.3 inj/1000 netball players	All
Pringle et al. (1998) [61]	NZ	Cross-sectional study: Trained observers recorded rugby union, rugby league & netball inj data. Standardised incident form, follow-up telephone calls by Physio’s monitored to recovery	Not known 4 wk period of 1 season	Junior recreational level. 1512 netball players, 15 inj Age 5–16 y	All inj that impaired a player’s performance. Severity classified on time-lost as minor or moderate	Rate: 13inj/1000 player hrs. Exposure: Not clear	All
Hume & Steele (2000) [62]	AUS	Prospective cohort study: State competition: 94 teams. Questionnaire completed by inj player reporting for medical treatment during	1995 3-day champs	Sub-elite level: U17, U19, U23 & Open (over 23 y). 940 netball players, 131 inj. Mean age 14.4 ± 4.4 y	All players reporting for treatment of any inj incurred during the 3-day champs	Rate: 139.4 inj/1000 players; 23.8/1000 playing hrs Exposure: Estimated individual player match hrs	All
Stevenson et al. (2000) [14]	AUS	Prospective cohort WASIS study: Baseline data and incidence of injury, self-report questionnaire with follow-up telephone interview once/m over season	1997 1 × 5m season	Community level. 258 netball players, 112 inj Age 9–56 y, mean 22 y	Council of Europe definition: any inj occurring during sports participation leads to: reduction in sports activity, need for advice/treatment and/or adverse economic or social effects. Severity based on level of treatment graded minor, moderate or severe	Rate: 12.1 inj/1000 h of participation Exposure: Mean individual combined match and training hrs	All
Finch et al. (2002) [15]	AUS	Prospective cohort WASIS study: Baseline data and inj incidence; self-report questionnaire with follow-up telephone surveys once/m over season	1997– 1998 2 × 5 m seasons	Community level 247 netball players, 216 inj Mean age 22 ± 8 y	Council of Europe definition: see Stevenson et al. (2000) Severity based on level of treatment graded minor, moderate or severe	Rate: 11.3 inj/1000 h of participation Exposure: Mean individual combined match and training hrs	All
Finch & Cassell (2006) [63]	AUS	Retrospective cohort study: Self-report household telephone survey of sports & active recreation inj	Not known Previous inj every 2 wk over 12 m	Community/ recreational level. Total 1084 participants; 648 across all sports, 34 net inj. Age 5 y +	Any inj during sport or active recreation regardless of treatment or time loss. Significant injury: required treatment, interfered daily activity &/or impacted subsequent activity	Rate: 19 inj/10,000 population; 51/1000 participants	All
Langeveld et al. (2012) [19]	SA	Prospective cohort study: USSA & National champs. Questionnaire completed by team manager, coach or medical staff daily	2009 3 × champs 4–6 days	Elite/Sub-elite U19, U21 & Senior players. 1280 netball players, 205 inj Severity: No missed matches	Any physical complaint during a netball match or training requiring medical attention. Severity: no. missed matches. Recurrent inj: same type as index inj post recovery from index inj	Rate: 500.7 inj/1000 playing hrs. Exposure: Individual player match time (mins) before inj	All
Pillay & Frantz (2012) [64]	SA	Cross-sectional study: Self-report questionnaire of player inj collected at a tournament	2010 1 × previous season	Elite/Sub-elite level. Total 254 players, 301 inj Age: 55 Club 24.1 ± 6.3 y; 147 Provincial 23.9 ± 5.1; 52 National 24.3 ± 4.3	Any physical complaint during match or training irrespective of need for medical attention or time loss. Severity based on symptoms graded as mild, moderate, severe. Repeated inj: inj to same site	Rate: 1.9 inj/player/ season	All
Singh et al. (2013) [65]	JAM	Retrospective cohort study: Self-report questionnaire of player inj & related inj factors	2003 –2007 Previous inj 5 y	Elite/Sub elite players: Senior (over 21 y), U21 & U16 age groups. Total of 59 players, 70 inj	Trauma to body resulting in the cessation of play. Severity: no definition used Grade I, II or II. Recurrent inj: repeated inj to same site	Proportion: 68% players inj	All
Ellapen et al. (2015) [66]	SA	Retrospective cohort study: Province School League: 80 Schools. Self-report questionnaire of netball inj history & related inj factors	Not known Previous inj 12 m recall period	Junior (school) level. Total 413 players, 258 inj Age 13–17 y	Distress or pain while playing netball preventing physical activity for > 1 day. Pain severity rating 1–5: no pain, mild, moderate, severe, worst pain	Proportion: 62% of players inj	All
Bissell & Lorentzos (2018) [67]	AUS	Prospective cohort study: 1 club. Self-report questionnaire on overuse inj (Oslo Sports Trauma Center questionnaire). Recorded 1 × per wk	Not known 1 × 12 wk season	Recreational/Club level players. Total 37 players, 152 overuse inj cases in 42 players. Age: adults < 45 y	All players reporting overuse inj during the 12 wk season. Severity score 0–25: based on time-loss and pain	Inj prevalence: 77.7%. 25% significant overuse inj	Overuse inj of knee, ankle & shoulder
Smyth et al. (2020) [68]	AUS	Prospective cohort study: ANNC competition: 16 teams. Medical attention & self-report inj data	2018 6-day champs	Sub-elite level netball players. Total 192 players; 96 U17, 96 U19. 103 inj	Concurrent IDCF: Any inj that required physio assessment irrespective of timeloss. Sports incapacity: inj resulted in any match time-loss or reduction in capacity based on HPQ Injury Classification: OSIICS	Rate: 89.4 inj/1000 player hrs. Sports incapacity: 19.1 inj/1000 player hrs. Exposure: Individual athlete & mean team match exposure (no athletes x no. teams x matches x min/match)	All
Botha et al. (2020) [69]	SA	Cross-sectional study: 2 junior tournaments. Standardised self-report questionnaire on inj & training modalities	2015 + 2017 Duration not stated	Junior (U15, U16) & Senior (U19) school level netball players. Total 560 players, 46 inj: 220 U15, 17 inj; 220 U16, 20 inj; 120 U19, 9 inj	Any physical complaint during netball match-play or training requiring medical attention	Rate: total 22.5 inj/1000 playing hrs. U15: 22.8; U16: 22.8; U19: 21.2. Exposure: Mean team match playing hrs (no. matches x game length x players)	All
Sinclair et al. (2020) [70]	SA	Cross-sectional study: School & State leagues. Inj diagnosed by doctor. Inj questionnaire completed with support of research assistant weekly	2017–2018 2 × seasons	U18 secondary school, U19, U21 & Senior Free State netball players. Total 96 players, 48 inj	Any physical complaint during netball match-play or training requiring medical treatment, loss of time or performance restriction. Severity: based on time-loss categories slight, minor, moderate, major	Rate: 33.9 inj/1000 h of match play Exposure: Mean match hours (1 match = 14 playing hrs)	All
Janse van Rensburg et al. (2021) [18]	SA	Prospective cohort study: Netball World Cup 2019: 16 teams. Inj surveillance forms completed by team physician &/or venue doctors	2019 10-day champs	Elite level players. 16 national teams. Total 192 players, 49 inj Mean age 26.6 y (95%CI: 25.9–27.3)	Any newly acquired inj & exacerbations requiring medical attention during the tournament. Severity: number days lost Recurrent inj: recovery from index injury and subsequent presentation of same inj	Rate: 54.8/1000 player hrs. Prevalence 20.3% Exposure: Mean individual match hrs (1 h × 7 players = 7 match player hrs/team/match)	All
Toohey et al. (2022) [71]	AUS	Prospective cohort study: SSN competition: 8 teams. Inj data collected during preseason, in-season & offseason. Data recorded by doctor or Physiotherapist using centralised database	2017 – 2019 3 × 17 wk seasons	Elite level players. 8 teams, total 119 players, 866 inj. Mean age 25.4 ± 4.2 y	All inj requiring medical attention and time-loss. Severity based on length of time-loss. Injury burden: mean severity x inj incidence Subsequent injury: any inj following initial inj in time period Injury Classification: OSIICS	Rate: 3.9 inj/365 player days. Exposure: Pre-season & in-season team hrs based on player contract days (no. contracted players x no. teams x no. surveillance days)	All
Kumari & Chaudhary (2023) [72]	IND	Prospective cohort study: All India inter university tournament. 52 teams Data recorded at the Central University of Haryana health centre	Not known	University level players. Total 14 players injured. Age range 18–24 y	Inj involving foot pain, finger pain, finger cuts, leg pain, and ankle twist referred to University health centre	Not provided: 3 finger pain; 7 leg pain; 2 ankle twist; 1 finger cut; 1 foot pain	Foot, finger, leg, ankle

*AUS* Australia, *NZ* New Zealand, *SA* South Africa, *JAM*, Jamaica, *UK* United Kingdom, *IND* India, *Inj* injury/injuries, *Physio* Physiotherapy/Physiotherapist, *Champs* Championships, *y* year, *m* month, *wk* week, *hrs* hours, *Av* Average, *no.* number, *U* Under, *SD* standard deviation, *AIS* Australian Institute of Sport, *NISU* National Injury Surveillance Unit, *WASIS* Western Australian Sports Injury Study, *ANNC* Australian National Netball Championships, *IDCF* Concurrent Injury Definitions Concept Framework, *HPQ* Oslo Sports Trauma Research Centre Health Problems Questionnaire, *SSN* Suncorp Super Netball League

**Table 3:** Methodological details and results of netball analytic epidemiological studies

Study	Country	Study design & data collection methods	Data collection period	Population	Injury definitions	Injury proportion or injury rate & exposure	Body Region	Risk factors
Hopper & Elliott, (1993) [73]	AUS	Retrospective & Prospective cohort study: National champs. Questionnaire of inj history & inj data recorded during champs. Risk factors measured at start of champs	1988 Multi-day champs	Elite/Sub-elite level: U16, U21 & Open (over 21). Total 228 players, 52 inj. Mean age: U16: 14.8 y, U21: 19.2 y, U21 23.7 y	A lower limb or back disability that caused pain or some form of dysfunction. Severity based on deformation grades 1–3	Proportion: 23% sustained lower limb or back inj	Lower Limb & Back	Age, previous inj, inj side, weak joints, lower limb and back podiatric variables: foot types & hip extension & external rotation (back problems), level of comp, taping/bracing, quarter & time in quarter
Hopper et al. (1994) [74]	AUS	Retrospective cohort study: National Champs Questionnaire of inj history prior to champs. Risk factors measured during champs	1988 Multi-day champs	Elite/Sub-elite level: U16 & U21. Total 204 players, 188 inj. Mean age U16: 14.8 U21: 19.1 y	All previous lower limb injuries	Proportion: 90% lower limb inj in career	Lower Limb	Previous inj, inj side, foot type
Hopper et al. (1995b) [75]	AUS	Prospective cohort study: State competition: 8 states Questionnaire completed by players & physio post treatment during 14-wk State comp. Risk factors recorded pre-season	1989 1 × 14 wk season	Elite to recreational level. 72 Senior players, 22 inj Age 15–36, mean 20.6 ± 3.6	Any inj presenting to first aid room requiring immediate medical care or resulting in some form of disability. No minor injuries. Severity based on deformation grades 1–3	Proportion: 30.6% players inj	All	Age, ht, mass, somatotype, hypermobility, static balance, muscular power, anaerobic fitness, level of comp, time-loss, treatment required, referral type
Hopper (1997) [76]	AUS	Prospective cohort study: National champs Lower limb and back inj diagnosed, treated & recorded by physio during champs. Risk factors measured pre-season	1988 Multi-day champs	Elite/Sub-elite. Total 213 U16, U21 & Open players, 52 inj. Mean ages: U16 14.8 ± 0.4, U21 19.2 ± 2.2, Open 23.7 ± 3.6 y	A lower limb or back disability that caused pain or some form of dysfunction	Proportion: 24% sustained lower limb or back inj	Lower Limb & Back	Age, playing position, somatotype, level of comp,
Smith et al. (2005) [77]	AUS	Cross sectional study: NSW Junior League Questionnaires of players self-reported inj. Risk factors measured during early season	All previous inj	Junior level. Total 200 players from 13 clubs, 69 injuries. Age 6- 16, mean 11 ± 2.5 y	Trauma to body part causing player to cease play & miss minimum 1 game	Proportion: 35% of players inj playing netball	All	Age, ethnicity, playing position, previous netball inj, other sport inj, playing experience (y), no. games/week, protective equipment, hypermobility (Beighton) score
McManus et al. (2006) [16]	AUS	Prospective cohort WASIS study: Risk factors and injury incidence from 2 consecutive 5 m seasons, baseline questionnaire and monthly telephone interviews	1997–1998 2 × seasons	Community level. Total 368 players, 272 inj. Age 66% 16–30 y	Inj during sport causing reduction in activity, need for medical advice &/or adverse social or economic effects. Recurrent inj: repeated index inj post recovery	Rate: 14 inj/1000 player hrs Exposure: Individual combined match and training hrs	All	Previous inj history, playing experience (y), time in season, training in previous y, pre-season training, training/wk, warm-up/cool-down, open to new ideas
Ferreira & Spamer (2010) [78]	SA	Prospective cohort study: Injuries recorded by physio at clinic. Risk factors recorded pre-& post season	2007 1 × season	Elite North-West University first team. Total 25 players, 46 inj. Age 18–23 y	All inj during match or training activities. Severity based on time-loss graded 1, 2 or 3	Rate: 1.84 inj/ player	All	Time in season, Anthropometrics: ht, mass, BMI, body fat %; Biomechanics: symmetry, dynamic mobility, local stability of limb-pelvic region, hip girdle, lower limb (knee and foot); Physical ability: agility, balance, explosive power
Maulder (2013) [79]	NZ	Prospective cohort study: Inj self-reported every 2 weeks via email/phone. Risk factors recorded preseason	Not Known 1 × 6 m season	Elite and Subelite level. Total of 24 players, 9 inj Age 18–25, mean 21.6 ± 3.2 y	All lower limb inj that affected performance & required medical treatment, causing missed training &/or game time	Proportion: 37.5% of players inj	Lower Limb	Lower limb dominance & asymmetry, agility performance: unanticipated straightrun & 180° turn tasks
Coetzee et al. (2014) [80]	SA	Prospective cohort study: USSA & National champs. Questionnaire of injuries & training history modality, completed by team manager, coach or medical staff daily during champs	2009 3 × champs 4–6 days	Elite level. U19, U21 & Senior. Total of 1280 participants, 205 inj	Same as Langeveld et al. 2012	Rate: 500.7 inj/1000 playing hrs Exposure: Individual player match time (mins) before inj	All	Training volume, training type (core stability, neuromuscular, biomechanical & proprioceptive training), playing surface
Attenborough et al. (2016) [81]	AUS	Cross-sectional study: Recurrent ankle sprain history collected preseason via self-report questionnaire. CAI measures: perceived & mechanical ankle instability	2013–2014 1 × season	Elite/inter-district & club level. 42 Club, 54, Elite/inter-district: total 96 players, 69 inj. Mean age: 21.5 ± 6.3 y	CAI: recurrent ankle sprain &/or perceived ankle instability &/or mechanical ankle stability. Severity: CAIT-Y score Recurrent sprain: 2 or more sprains to same ankle	Proportion: 72% previous ankle sprain, 47% recurrent sprain	Ankle	Previous inj, static & dynamic balance (SEBT), age, ht, mass, level of competition,
Stuelcken et al. (2016) [82]	AUS	Retrospective study: ANZ champs Medically diagnosed, televised ACL injuries. Inj mechanisms identified from video	2009–2015 Televised games 6 y 3m	Elite level. Total of 16 players, 16 ACL inj. Age not reported	All televised ACL injuries during ANZ champs	Proportion: 63% left knee, 37% right knee	Knee	Game situation, movement patterns, player behaviour & potential mechanism at time of injury, playing position, match quarter
Attenborough et al. (2017) [83]	AUS	Prospective cohort study: Ankle inj & exposure data collected by team physio or via self-report. Risk factor data collected pre-season	2013–2014 1 × season	Elite/inter-district & club level. Total 94 players, 11 inj. Mean age: 21.5 ± 6.3 y	All ankle injuries resulting in time loss ≥ one full match or training Session Severity: CAIT-Y score	Rate: 1.74/1000 h; 6.75/1000 h matchplay; 0.40/1000 h training Exposure: Individual player recorded match & training hrs	Ankle	Perceived ankle instability, ankle sprain history, joint laxity, muscular power, static & dynamic balance (SEBT), age, ht, mass, level of competition,
Pickering Rodriguez et al. (2017) [84]	AUS	Prospective cohort study: National & State champs. Lower body overuse inj data reported by physio or self-report. Risk factor data collected preseason &1 x/month across season	2013 1 × 14 wk season	Elite & sub-elite level. Total 29 players, 12 inj. Mean age 24.1 ± 3.2 y	Non-contact, match or training, soft tissue damage of lower limb resulting in time loss ≥ 1 game	Rate: 11.29/1000 h; Elite: 19.35/1000 h; Sub-elite:7.13/1000 h Exposure: Team combined match & training hrs	Lower Limb	Lower body stiffness age, ht, mass, level of competition,
Whatman & Reid (2017) [85]	NZ	Cross-sectional study: Self-report overuse knee & ankle inj history (Oslo Sports Trauma Center questionnaire). Risk factor data collected during tournament	Not Known Previous inj 12 m	Junior Secondary School level. Total 166 players, mean age 16 ± 1 y	All ankle & knee inj with no identifiable event responsible for onset. Substantial inj: moderate or severe reduction in or inability to compete in matches or training	Prevalence Knee: 31%, Substantial inj: 10%; Ankle: 51%, Substantial inj 24%	Ankle & Knee	Previous inj, level of play, movement competency: dorsiflexion ROM, frontal-plane knee angle + position during single-leg squat & drop jump, vertical jump ht & power
Horgan et al. (2020) [86]	AUS	Retrospective cohort study: National Secondary School tournament. Inj & risk factor data collected form self-report and medical diagnosis, recorded on AIS AMS	2015–2018 4 y	Elite & Pre-elite level. Total 536 players, 1122 inj. Mean age 18.8 ± 4.6 y	Loss or abnormality of bodily structure or functioning during training or competition diagnosed as a Medically recognised inj	Daily probability 0.98 ± 0.06%	All	Training preparedness (fatigue, mood, motivation, soreness, sleep duration & quality), training load, time following inj
Franettovich Smith et al. (2020) [17]	AUS	Prospective cohort study: 1 club playing across 9 divisions. Inj recorded by player/coach. Follow-up telephone call from researcher. Risk factor data recorded pre-season via questionnaire	2016 1 × season	Community/ recreational level. Total 269 players, 169 inj. Age 7–42 y	All lower limb bodily damage caused by competing or training for netball	Rate: 13.8/1000 h. Match: 32.3, Training 4.7/1000 h Exposure: individual player recorded match & training hrs	All	Age, ht, mass, BMI, previous inj, netball hrs/wk, other physical activity hrs/wk, use of warm-up & cool-down, taping or bracing, footwear, ankle dorsiflexion ROM, level of comp, time in season, season game time, training time
Sinclair et al. (2021) [87]	SA	Prospective cohort study: Self-administered inj report questionnaire, weekly follow-up. Risk factor data recorded preseason	2017–2018 1 × season	U18 secondary school, U19, U21 & Senior Elite Free State level. Total 110 players, 48 inj	Same as Sinclair et al. 2020	Rate: 33.9 inj/1000 h of match play Exposure: Team mean match hours (1 match = 14 playing hrs)	All	Age, playing position, previous inj history, ht, wt, BMI, body fat, balance, flexibility, explosive power, upper & lower body strength, core strength, speed & agility
Belcher et al. (2022) [88]	NZ	Cross-sectional study: ANZ or International comps. Systematic video analysis of medically diagnosed, televised ACL inj	2011–2019 8.5 y	Elite level. Total 21 players with ACL inj Age not reported	All televised ACL inj during matchplay	Proportion: 57% left, 43% right knee	Knee	Game situation, movement patterns, player behaviour, inj mechanism at time of injury, inj side, playing position, match quarter
Mullally et al. (2023) [89]	UK	Cross-sectional study: Online survey; self-report inj previous 12 m, and risk factors. Administered worldwide	Not Known Previous inj 12 m recall period	Recreational level. Total 193 players, 73 upper limb, 182 lower limb inj. Age > 18 y, mean 33.7 ± 11.2 y	Any netball inj sustained in previous 12 m, & knee inj in previous 5 y, that prevented participation in ≥ 1 match or training session	Rate: Upper limb: 37.8 inj/100 players/y Lower limb: 94.3 inj/100 players/y	All	Injury situations, previous inj, playing position, match or training inj, match quarter, time-loss, treatment type,
Hammill (2024) [90]	SA	Cross-sectional study: Inj data collected biweekly using online inj questionnaire supported by qualified field workers. Risk factor data collected at beginning of season	Not Known 1 × season	University level. Total 17 players, 10 inj. Mean age 20.8 ± 1.4 y	All lower extremity injuries	10 lower extremity inj	Lower Limb	Age, ht, mass, body fat %, isokinetic knee strength, quadricep: hamstring ratio, inj side
Jolingana-Seoka et al. (2024) [91]	SA	Cross-Sectional study: Self-report inj data collected bi-weekly via online questionnaire. Risk factor data collected preseason	2022 1 × season	University level netball players. Total 10 players. Mean age 21.2 ± 1.4 y	All lower extremity injuries	Total unknown Proportion: 30% ankle, 20% foot, 20% back, 10% knee, 10% calf, 10% hip inj	Lower Limb	Ankle ROM, isokinetic strength, lower limb muscle activity, limb dominance

*AUS* Australia, *NZ* New Zealand, *SA*, South Africa, *Inj* injury/injuries, *Comp* Competition, *Physio* Physiotherapy/Physiotherapist, *Champs* Championships, *y* year, *m* Month, *wk(s)* week(s), *U* Under, *ht* height, *wt* weight, *BMI* body mass index, *SD* standard deviation, *WASIS*, Western Australian Sports Injury Study, *CAI* Chronic Ankle Instability, *CAITY* Cumberland Ankle Instability Tool, *AIS* Australian Institute of Sport, *AMS* Athlete Management System, *ANZ* Australia and New Zealand premiership, *RTS* Return to Sport, *ConQ:ConH ratio* concentric quadriceps: concentric hamstring ratio, *ROM* range of motion

### Year of Publication

Eighteen descriptive epidemiology studies were conducted pre–2008 (41%), 14% of which reported data from pre–1998 [30, 34–36, 59, 60]. Post 2018, eight hospital/clinic record studies (18%) [51–58], and six (14%) netball competition studies [18, 68–72] have been conducted. The analytic research has increased considerably in the 15 years since 2008 (*n* = 15, 71%), with nearly half of these studies conducted since 2019 [17, 86–91]. Additional File 2, Table 1 presents the frequency of studies according to publication year. It is also important to note that all of the studies report injury data from a minimum of 1 year [34], up to a maximum of 16 years [36] prior to the publication date.

### Country of Origin

Eight of the 77 netball countries affiliated to World Netball [1] have conducted injury research. Most studies were conducted in Australia (*n* = 32, 49%), New Zealand (*n* = 14, 22%) and South Africa (*n* = 11, 17%). Australian studies focused on descriptive studies of netball competitions [14, 15, 30, 59, 60, 62, 63, 67, 68, 71] and analytical studies [16, 17, 73–77, 81–84, 86]. In contrast, New Zealand largely utilised hospital/clinic and insurance data [35, 36, 38, 44, 46–48, 52, 54, 55]. South African studies assessed both descriptive studies of netball competitions [18, 19, 64, 66, 69, 70], and analytic studies [78, 80, 87, 90, 91]. Only four (6%) injury studies, comprising three hospital/clinic and insurance studies [39, 53, 57] and one analytic study [89], have been conducted in the UK, with no netball competition studies to date (Tables 1, 2 and 3, Additional File 2, Fig. 2).

### Data Collection Period

A wide range of data collection periods were used across the netball studies with hospital/clinic or insurance data reporting the longest periods (Table 1). Most studies collected data for 4 years or more (*n* = 14, 56%) [36–38, 42, 44–49, 51, 52, 55, 58], or periods lasting 1 year (*n* = 9, 36%) [35, 36, 39–41, 43, 50, 54, 57], 2–3 years (12%) [35, 53, 56], or 10 months [34].

Descriptive netball competition studies collected data during netball seasons (*n* = 8, 42%), netball tournaments (*n* = 6, 32%) or over time periods (*n* = 5, 26%). The season data included studies assessing specific state or school leagues over one 14-week season [30], two seasons [60, 70], three 17-week seasons [71] and five 14-week seasons [59]. Other studies assessed injuries in players across one five-month season, two five-month seasons [14, 15] or one club during one 12-week season [67]. Studies assessing tournaments collected data for 3 days [62, 64], 4–6 days [19], 6 days [68] and 10 days [18], while those analysing time periods included 12 months [63, 66], 4 weeks of 1 season [61], one previous season [64], and 5 years [65].

The analytic studies similarly recorded injury data over seasons (*n* = 11, 52%), tournaments (*n* = 4, 19%) or time periods (*n* = 6, 29%). Season data assessed state leagues lasting one 14-week season [75, 84], injured players over one season [78, 79, 81, 83, 87, 90, 91] or two seasons [16] and one club over one season [17]. Other studies reported injury data from multi-day tournaments [73, 74, 76, 80], or time periods including the previous 12 months [85, 89], 4 years [86], 6 years 3 months [82] and 8.5 years [88]. One study collected data on all pervious injuries [77].

### Study Populations

The populations investigated across the netball injury studies showed considerable variation. The hospital/clinic and insurance studies (Table 1) had the largest number of participants, ranging from 3 [40] to 11,757 [52], with 60% including > 100 participants [35–38, 43–46, 49–54, 58], and 40% > 1000 [37, 44–46, 49–52, 54, 58]. Most studies included a combination of children and adult age groups (60%) [35, 37, 38, 40, 41, 43–47, 49, 50, 53, 55, 58], with children typically categorised as under 15 years (y). A further seven (28%) studies analysed adults (15 y +) [34, 36, 42, 48, 51, 54, 56], while Hassan & Dorani [39] assessed children between 5–15 y.

The netball competition studies (Table 2) analysing season long competitions, included populations ranging from 37 [67] to 11,228 [59], with 56% (*n* = 5) < 300 participants [14, 15, 67, 70, 71]. The populations consisted of adults and children in four studies [14, 15, 30, 59], adults in three studies [60, 67, 71], while typical netball age categories; under 18, 19 and 21 were used by Sinclair et al. [70]. In studies assessing tournaments, populations ranged from 14 [72] to 1280 [19], with 50% < 200 [18, 62, 68, 69, 72]. Two studies analysed adults [18, 72], with four assessing a combination of adult and junior age categories (under15 to under 21 and senior level) [19, 62, 68, 69]. The four studies analysing time periods included populations ranging from 59 [65] to 1512 [61], with 50% > 1000 [61, 63]. Participants included junior [61], junior school [66], children and adult age groups [63] and under 16, 21 and senior age categories [65].

The analytic studies reported the smallest populations (Table 3). Those analysing seasons included cohorts ranging from 10 [91] to 368 [16] participants, of which 81% included populations of < 100 adult participants [16, 75, 78, 79, 81, 83, 84, 90, 91]. Tournament study populations ranged from 204 [74] to 1280 [80] participants, of which 75% had < 300, including under 16, under 21 and Open (adult) participants [73, 74, 76]. The six studies analysing time periods assessed populations of 16 [82] to 536 [86] athletes, typically < 200 (67%) [82, 85, 88, 89], two of which assessed the ACL injury mechanisms of elite athletes [82, 88].

### Level of competition

The hospital/clinic and insurance studies (Table 1) mostly assessed the general population across all levels (68%) [37–42, 46–50, 52–54, 56–58] or netball populations across all levels (28%) [35, 36, 43–45, 51, 55], with one study investigating elite netball [34]. Specific competition levels or a combination of levels were more frequently analysed in netball competition and analytic studies (Tables 2 and 3). Studies analysing netball competitions assessed a combination of levels in six (32%) studies, reported as elite & sub-elite [19, 64, 65], elite & recreational [60] and recreational to competitive levels [30, 59]. Studies in this category also assessed players at the recreational/community (club) level [14, 15, 63, 67], junior and senior school level [69, 70], elite level [18, 71], sub-elite level [62], recreational junior level [61] and university level [72]. The analytic studies similarly assessed a combination of elite and sub-elite levels (29%) [73, 74, 76, 79, 84, 86], recreational/community (club) level [16, 17, 77, 89], university level [78, 90, 91] and elite level netballers [80, 82, 88]. Of the studies conducted at the elite level two analysed the Australia and New Zealand premiership (ANZ) [82, 88], one investigated the Netball World Cup [18], and one the Suncorp Super Netball competition [71].

### Data Collection Methods

The methods of data collection in the hospital/clinic and insurance studies all involved diagnosis of injuries by medical professionals. In contrast, the netball competition and analytic studies used a wider range of data collection methods (Tables 2 and 3; Additional File 2, Fig. 4). Data was collected via player self-reporting of injuries in 47% of netball competition [14, 15, 62–67, 69] and 38% of the analytic studies [16, 74, 79, 81, 85, 87, 89, 91]. A combination of self-reporting and medical professional diagnosis also in combination with the coach/manager was used in 32% of netball competition [19, 30, 59–61, 68] and 43% of analytic studies [17, 73, 75, 77, 80, 83, 84, 86, 90]. Medical professionals, typically physiotherapists, diagnosed player injuries in 21% of netball competition studies [18, 70–72] and 19% of analytic studies [76, 78, 82, 88]. The data collection methods used in the netball competition and analytic studies were influenced by the level of competition, with medical professional diagnosis typically used at the elite level (80%) [18, 71, 82, 88] and self-report at the recreational/community level (75%) [14–16, 63, 67, 89].

Across the netball competition and analytic studies, only two netball injury studies captured longitudinal data of all injuries from ongoing and systematic injury surveillance systems. Toohey et al. [71] reports standardised injury data from a cohort of elite players in the Suncorp Netball Superleague, assessing 119 players from 8 teams across three seasons using the Australian Institute of Sport (AIS) customised Athlete Management System (AMS) database. Horgan et al. [86] also report 4 years of retrospective data from the same centralised database in a cohort of 536 elite and pre-elite athletes.

### Body Regions

Most netball injury studies assessed injuries across all body regions (60%), shown in Tables 1, 2 and 3 and Additional File 2, Fig. 3. The most common specific body regions analysed were the knee and lower limb. Five (20%) hospital/clinic and insurance studies [42, 46, 49, 54, 56], and two analytic studies [82, 88] assessed the knee. Five (24%) analytic studies focused on the lower limb injuries [74, 79, 84, 91, 92], while 2 assessed lower limb and back injuries [73, 76]. Two further analytic studies assessed ankle injuries [81, 83]. The hospital/insurance data studies also assessed fractures across all body regions [39, 40, 57], dental injuries [38, 47], and Achilles Tendon injuries [48].

### Injury Definitions

A wide range of injury definitions were used in the netball injury research (Tables 1, 2 and 3). Hospital/clinic or insurance studies used medical attention definitions in 44% of studies, referring to clinic or hospital attendance [34–37, 41, 44, 45, 50, 52, 53, 58], while 28% used medical attention definitions related to specific injuries; fractures [39, 40, 57]; ACL [42, 49, 56]; Achilles Tendon [48]. A further 28% included any complaint resulting in an insurance claim, in relation to all injuries [43, 51], dental injuries [38, 47] and ACL injuries [46, 54].

Netball competition studies used any or all complaints definitions in 58% of studies; five used any complaints that impaired performance [60, 61, 63, 64, 67], three any complaints leading to medical attention and time-loss [18, 70, 71] and three approved sports injury definitions [14, 15, 68]. Six studies (32%) used medical attention definitions [19, 30, 59, 62, 69, 72], two of which excluded minor injuries [30, 59] and time-loss from training or competition definitions was used in two studies [65, 66]. The analytic studies used all complaints definitions in six studies (38%) [17, 74, 78, 87, 90, 91] and medical attention and time-loss in two studies [16, 79]. Medical attention definitions were used in five (24%) studies [73, 75, 76, 80, 86], time-loss criteria in five studies (24%) [77, 83–85, 89], and definitions relating to specific injuries in three studies [81, 82, 88]. A small proportion of studies identified injuries as new or recurrent (*n* = 11, 17%). The term recurrent injury was mostly used and defined as the same injury as an index injury post recovery [16, 18, 19, 65, 80]. Subsequent injuries were defined by Toohey et al. [71] as any injury, following an initial injury in the time period.

### Injury Severity and Burden

Injury severity definitions were reported in 40% of the hospital/clinic and insurance studies. Four studies used recognised injury severity scoring systems [35, 36, 39, 44], others reported the number or type of treatment [34, 57] and proxy measures based on the cost of injury [43, 52] or admission/length of stay in hospital [37, 41]. Fourteen (56%) of the netball competition studies reported injury severity, of which 50% used time-loss from participation definitions [18, 19, 60, 61, 68, 70, 71]. Other studies defined severity based on injury symptoms [59, 64], level of treatment [14, 15], treatment and time-loss combined [63] or pain ratings [66, 67]. Similarly, most analytic epidemiology studies reporting injury severity (38%) used time-loss definitions [78, 80, 85, 87] or specific injury scoring tools [81, 82] (Tables 1, 2 and 3). Severity ratings across the studies were typically based on grades or categories, either grades 1–3, or categories most commonly minor, moderate and severe. Only one study reported injury burden across the 65 included studies. Toohey et al. [71] defined burden as the product of mean severity and injury incidence.

### Injury Classifications

Injuries were typically classified across the studies by body location or the location and type of injury, but recognised injury classification systems were only used in nine studies (14%). The International Classification of Diseases (ICD) [92] was used in six hospital/clinic or insurance studies [41, 44, 45, 49, 53, 58] and the Orchard Sports Injury Classification System (OSIICS) [93] was used in one hospital/clinic or insurance study [51] and two netball competition studies [68, 71]. Injuries were additionally classified by the mode of onset (traumatic or overuse) in two hospital hospital/clinic or insurance studies [34, 52], seven netball competition studies [18, 19, 62, 67, 68, 70, 71] and three analytic studies [73, 80, 87].

### Injury Incidence rates

Tables 1, 2 and 3 show a small number of studies reported the total number of injuries only [52, 53, 72, 90], while others reported the proportion of injuries; 11 hospital/clinic and insurance studies [37, 39–42, 46–48, 54, 56, 57]; two netball competition studies [65, 66]; ten analytic studies [73–77, 79, 81, 82, 88,]. All other studies used a range of methods to report injury rates. The hospital/clinic and insurance studies typically used injury rates in relation to an actual or estimated population (*n* = 10, 40%); mostly including rates per 100,000 netball participants [35, 36, 44, 49, 50, 58] or 1000 participants [43, 45, 51, 55]. Netball competition studies mostly (47%) reported injury rates per 1000 player hours [14, 15, 18, 19, 61, 62, 68–70]. Other studies reported rates per 1000 [60, 62] or 10,000 players [63], per player per season [64], per 1000 players/match [30] or per 365 player days [71], while two used injury prevalence [18, 67]. Injury rates per 1000 player hours was the method reported in 29% of analytic studies [16, 17, 80, 83, 84, 87], while other methods included injuries per player [78], per 100 players per year [89] per player per year [83], daily probability [86] and injury prevalence [85].

### Athlete Exposure

A variety of methods were used to calculate incidence rates based on athlete exposure hours (Tables 2 and 3). Studies mostly estimated match and/or training hours based on the average duration (hours) of playing and training in the time period [14, 15, 18, 62, 68, 69, 84, 87]. Only two studies calculated exposure based on individual match and training attendance records [17, 83]. Of the ten (53%) netball competition studies reporting athlete exposure, six used match exposure hours only [18, 19, 62, 68–70], with two combining match and training hours [14, 15]. Estimated individual exposure hours were determined in six studies [14, 15, 18, 62, 68, 70], while three calculated team hours [68, 69, 71]. The analytic studies (*n* = 6, 29%), utilised combined match and training hours in four studies [16, 17, 83, 84], and match hours in two [80, 87]. Individual exposure hours were used in three studies [16, 17, 80, 83] and team hours in two [84, 87]. Two further studies measured individual athlete exposure as the individual player match time in minutes before the injury occurred [19, 80].

### Injury Mechanisms

The mechanism or event causing an injury was identified in seven (28%) hospital/clinic or insurance record studies, reporting injury events in categories including: overexertion, falls and collisions [35, 36, 39, 42, 44, 46, 48]. Eleven (58%) netball competition studies [18, 30, 59–62, 64–66, 68, 70] described injury mechanisms. The injury questionnaires used in these studies provided common injury cause options including: sharp twists/turns, falls, incorrect landing, collision with player, trip/slip, trodden on foot, sudden stopping, struct by player/ball, overexertion or other reasons. Hopper et al. [59] and Hume & Steele [62] provided further detail including the playing strategy (attack or defence), playing action e.g. intercepting, and movement e.g. shuffling, at the time of injury. Eight (38%) analytic studies reported mechanisms as part of their injury analysis [73, 75, 78, 80, 83, 86, 87, 90]. Three further studies had a specific injury mechanism focus, including Mullally et al. [89] who assessed injury situations in relation to previous injury. Two studies assessed injury mechanisms using systematic video analysis methods providing a comprehensive assessment of the events leading to ACL injury [82, 88]. These studies provided detailed descriptions of the game situation, player movement patterns, player behaviour and qualitative biomechanics of netball injuries to identify patterns in ACL injury causes.

### Injury Risk Factors

The included studies have assessed a wide range of intrinsic and extrinsic risk factors and their association to injuries (Additional File 2, Table 2). The hospital/clinic or insurance studies assessed the smallest number of risk factors (median = 1, range 0–7 factors per study). The most common factors assessed were age (*n* = 12) [35, 39, 42–45, 48, 50–53, 55], gender (*n* = 8) [35, 40, 44, 45, 50, 52, 53, 58] and cost of injury (*n* = 3) [43, 50, 52]. Netball competition studies assessed a greater combination of risk factors (median = 4, range 0–11), with four studies analysing between 8 to 11 risk factors [30, 59, 65, 66]. The most frequent intrinsic factors assessed included age (*n* = 10) [14, 30, 62, 64–70], position (n = 8) [18, 19, 30, 64, 65, 69, 70, 72] and previous injury (*n* = 3) [15, 65, 68]. While the common extrinsic factors were weekly training (n = 8) [15, 30, 59, 60, 64, 68, 72], initial treatment required (*n* = 7) [15, 30, 59, 61, 64, 68, 72], training time (*n* = 6) [14, 30, 59, 65, 66, 68] and match quarter the injury occurred in (*n* = 6) [18, 19, 30, 59, 69, 70].

Commensurate with their purpose, the analytic studies assessed the widest range of risk factors (median = 6, range 3–15), with five studies assessing between 10 to 15 factors [17, 73, 75, 78, 87]. Table 3 and Additional File 2, Table 2 show the intrinsic factors most frequently analysed included age (*n* = 10) [17, 73, 75–77, 81, 83, 84, 87, 90], previous injury (*n* = 8) [16, 17, 73, 74, 77, 81, 83, 85, 87, 89], height (*n* = 8) and mass (*n* = 8) [17, 75, 78, 81, 83, 84, 87, 90], and playing position (*n* = 6) [76, 77, 82, 87–89]. A range of anatomical and biomechanical factors including limb dominance, postural stability, podiatric variables, ankle joint laxity and range of motion and lower body stiffness were assessed across 15 studies [17, 73–75, 77–81, 83–85, 87, 90, 91]. Physiological factors such as aerobic and anaerobic fitness, agility, strength, power, speed and flexibility, were additionally assessed across seven studies [75, 76, 78, 79, 83, 85, 87]. The extrinsic risk factors assessed included level of competition (*n* = 7) [17, 73, 75, 76, 81, 84, 85] and match quarter (*n* = 4) [73, 82, 88, 89] with a wide range of timing, training and treatment related factors also assessed across the 21 analytic studies.

### Data Analysis Methods

**Fig. 2:**
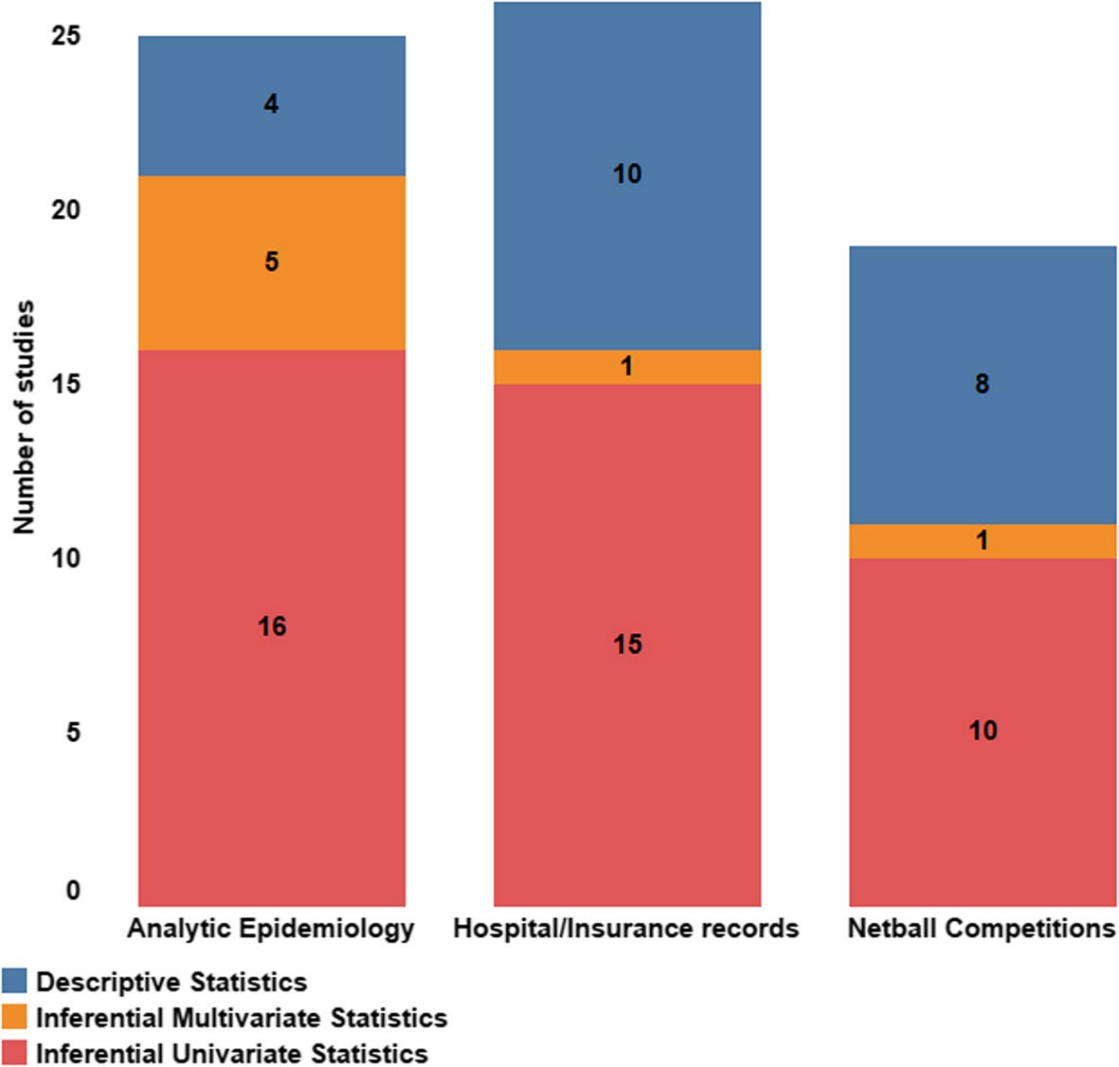
Frequency of Netball injury studies by study design and data analysis methods

The data analysis methods used across the studies included a range of descriptive and inferential statistics to describe the injury datasets (Fig. 2). Over 40% of the hospital/insurance records [34–37, 40, 41, 44, 46, 51, 58] and netball competition studies [19, 62–65, 69, 70, 72] reported descriptive statistics only. A small number of hospital/insurance record [38, 50, 55, 56] and netball competition studies [68, 71] reported odds ratios (injury probability), risk ratios (relative risk) or injury incidence rate ratios to describe differences between groups. Univariate inferential statistics were additionally used to assess the effect of various risk factors on injury in 60% of hospital/insurance record studies [38, 39, 42, 43, 45, 47–50, 52–57] and 53% of the netball competition studies [14, 15, 18, 30, 59–61, 66–68]. The chi-square test was the most frequent univariate test used in the descriptive studies (*n* = 19, 76%). Multivariate statistical tests were infrequent in these studies with only Fernando et al. [50] and Toohey et al. [71] using binary logistic regression models and generalised linear mixed models respectively.

Most analytic studies used inferential statistics to assess the effect of risk factors on injury (81%). Five studies used odds ratios [17, 77, 81, 84, 86], with risk ratios [84], absolute risk [86] and incidence rate ratios [16] also reported. Univariate statistics, including chi-square, t-tests, Mann–Whitney U tests, analysis of variance and univariate logistic regression, were used in 76% of studies [16, 17, 73–79, 81, 83–85, 87, 90, 91]. Five (24%) studies used multivariate tests, with all using multiple logistic regression models [17, 75, 77, 86, 87]. Adjustments for confounding variables was conducted in three studies [77, 86, 87]. The three studies with a focus on assessing injury mechanisms provided descriptive analysis only [82, 88, 89].

## Discussion

This scoping review presents the first comprehensive overview of research methodologies used to determine injury incidence, aetiology, and mechanisms in netball. It complements the recent reviews of netball injury research by Downs et al. [25] and Whitehead et al. [26], highlighting methodological considerations aligned with the first two steps of the van Mechelen et al. [20] and TRIPP [21] injury prevention models. A total of 65 netball injury studies were included following screening, consisting of 44 descriptive epidemiological studies and 21 analytic epidemiological studies. The review highlights a scarcity of studies using systematic and ongoing injury surveillance, as well as limited methodological approaches to assess injury aetiology and mechanisms in netball. Without a specific consensus statement for netball to guide injury research, this review proposes potential future directions to enhance the quality of the netball injury evidence base.

The extent of the injury problem in netball is described in the 44 descriptive epidemiological studies and 19 (90%) of the analytic studies reporting injury data. However, 41% of descriptive studies and 29% of analytic studies were published between 1986 to 2008, with injury data collected an average of 3.6 years prior to publication. Furthermore, the majority of netball injury research has been conducted in Southern Hemisphere countries (88%), predominantly Australia (49%), and thus does not represent all netball-playing nations. Recent advancements in injury data collection methods [94], together with the growing professionalisation of netball with its increased physical demands [26, 95], and variations in playing styles across countries [1], emphasise the need for further research. This should encompass the diverse range of playing nations to fully understand the injury problem in line with the demands of the modern game.

The netball injury research has utilised various data sources, including hospital, clinic, and insurance databases (39%), as well as different competition formats, and specified time periods (descriptive epidemiology 29%; analytic epidemiology 32%). While hospital/clinic or insurance studies, utilise large populations and longitudinal data [51], they primarily capture severe injuries [22, 25], thereby underestimating injury incidence by neglecting milder cases. In contrast, data from netball competitions capture a broader range of injuries, providing a more accurate portrayal of the sport’s injury problems. Yet, studies vary considerably in observation periods, including short tournaments of 3–10 days (25%), league competitions over single or multiple seasons (50%), or specified time periods (25%). The lack of netball injury studies reporting longitudinal data from ongoing, systematic injury surveillance systems is a key finding of this study. Ekergen et al. [22] emphasised the need for such systems to provide high-quality data for effective injury prevention. However, only two (3%) netball studies report injury data from “true” injury surveillance systems [22]. Toohey et al. [71] collected injury data from a prospective cohort, in the elite Suncorp Netball Superleague over three consecutive seasons, using standardised methods [94]. While Horgan et al. [86] assessed retrospective data from the same centralised database (AMS), to assess the impact of risk factors on previously recorded injuries. The lack of comprehensive injury surveillance impacts the accuracy and reliability of the current netball injury research.

The current netball injury studies employed diverse methodologies to collect injury data, utilising prospective, retrospective and cross-sectional designs across the study categories. Study populations included a broad range of netball participants ranging from 3 [40] to 11,757 [52], with many including a combination of age-groups and participation levels, often lacking clear definitions. Indeed, Ferreira & Spamer [78] defined “elite” netballers as University first team players, while Janse van Rensburg [18] defined “elite” as those representing their country at the 2019 Netball World Cup. Injury diagnosis methods also differed, hospital/clinic or insurance studies using medical professionals, while competition studies used mostly medical staff at the elite level (80%) and self-report methods at the community/recreational level (75%).

Injury definitions varied across injury studies, with hospital/clinic or insurance studies mainly employing medical attention definitions (72%), while competition and analytic studies used a broader range, including all complaints (51%), medical attention (30%) and time-loss definitions (19%). Definitions of injury severity also varied, incorporating time-loss, treatment, symptom, hospital attendance and cost of injury criteria. To date, Toohey et al. [71] is the only study to report injury burden, a critical measure that combines injury frequency with its severity (typically measured in days lost) [94]. This metric allows for the identification of not only the most common injuries but also those that impose the greatest impact [96]. This understanding is vital for comprehensively assessing the repercussions of injuries within netball. Furthermore, only a small number of studies defined recurrent injuries, (14%) or used a recognized classification system for injuries (14%).

The variations in study design and data collection methods make it difficult to compare netball injury studies, and differentiate injury risks within defined populations. The methodological issues subsequently impact the reported incidence rates in the current netball injury research. Moreover, the different metrics for calculating injury incidence further confuse the extent of the injury problem. Although more recent competition studies [14, 15, 18, 19, 61, 62, 68–70] and analytic studies [16, 17, 80, 83, 84, 87] report injuries in relation to athlete exposure hours, differences in exposure calculation methods, including using match hours only, combining match and training hours, and using average team or individual hours have also impacted the reported incidence rates. This has led to incidence rates ranging from 11.3 to 89.4 injuries/1000 player hours (Table 1, 2 and 3). Additionally, two further studies [19, 80] calculated player exposure based on game time in minutes prior to injury rather than total exposure time over the study period. This different approach to calculating athlete exposure resulted in a very high injury incidence rate of 500.7 injuries per 1000 h.

To develop a clear understanding of the injury problem [20, 21], robust injury surveillance systems are crucial for netball to ensure accurate data informs the evidence base. The England Rugby Football Union (RFU), has effectively implemented such systems across elite men’s and women’s levels (PRISP and WRISP projects), community level (CRISP project) and university level (BUCS ISP project) [97] providing an effective model for netball. Currently, no netball injury research has assessed the UK Netball Superleague, or New Zealand ANZ Premiership, and only one study assesses the Australian Suncorp Super Netball. Therefore, future research should focus on the development of robust surveillance systems to provide consistent injury data to analyse all competitions at the elite level. Furthermore, there is a need to develop tailored surveillance systems for all levels of the game.

This study recommends adopting the standardised methods of data collection in the International Olympic Committee (IOC) consensus statement [94] to ensure consistent surveillance methods. This updates the recommendations of Downs et al. [25], who endorsed the rugby union consensus statement [98]. The guidelines include consistent use of either all complaints, medical attention or time-loss injury definitions, and time-loss severity definitions, depending on the study focus. They suggest using measures of injury burden that combine frequency and consequences, typically injury incidence multiplied by severity (time-loss days). Recommendations for classifying injuries are provided using consistent coding systems such as the Orchard Sports Injury Classification System (OSIICS) [93]. Furthermore, to standardise the reporting of injury rates, the IOC statement recommends recording individual player exposure hours and expressing injury incidence rates per 1000 athlete exposure hours for sudden-onset injuries. For gradual-onset conditions, it suggests reporting prevalence as the proportion of injured athletes [94].

**Fig. 3:**
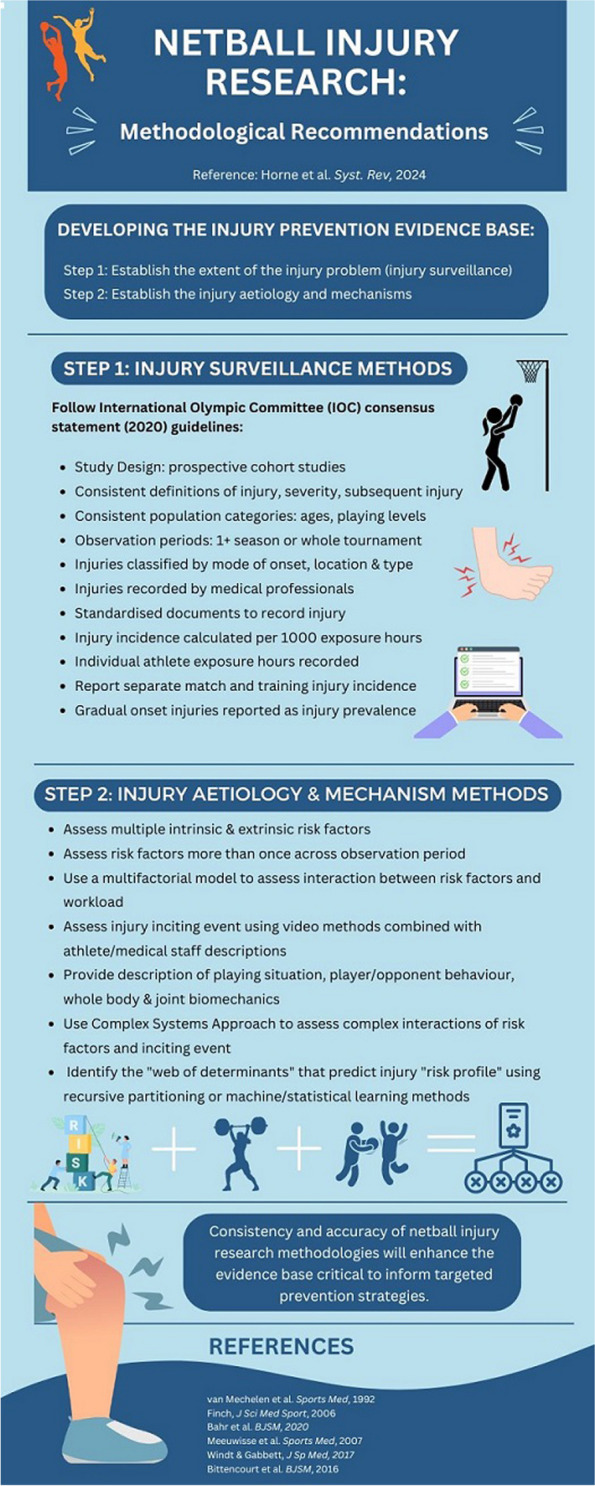
Netball injury research methodological recommendations

In addition to the IOC guidelines, this study advises incorporating netball-specific demographic categories to define study populations. Age categories such as Senior/Adult, under 21, under 19, under 17, and Junior levels such as Under 16, Under 15, Under 14 are universally used across nations in international, national, and school-level competitions, providing a consistent framework. Inclusion of age mean and range will further describe the age distribution within each category. To describe level of play we recommend classifying netball populations according to Mckay et al.’s. [99] skill level and training status framework. Participants are categorised using the criteria of Tier 0–4: Sedentary, Recreationally Active; Trained/Developmental; Highly Trained/National Level, Elite/International Level. In this framework Elite/International netball competitions would include all International competitions and elite leagues including the UK Netball Superleague, Suncorp Super Netball in Australia and ANZ Premiership in New Zealand. The consistent reporting of injuries using these categories would provide greater clarity regarding the injury issues across age groups and playing levels. A summary of guidelines to identify the injury problem, adapted for netball, are provided in Fig. 3.

The current research assessing injury aetiology and mechanisms in netball has notable limitations. Twenty-one analytical studies aimed to identify the factors causing injury, while a further 34 descriptive studies investigated isolated factors related to injury. Collectively, these studies have assessed a wide range of intrinsic and extrinsic risk factors, but typically only a small combination of factors within each study. Specifically, the analytic studies analysed a median of 6 risk factors across the 21 papers. Furthermore, most studies employed a reductionist approach, simplifying factors into units in a linear, unidirectional way. This approach is thought to restrict understanding of injury causes, particularly where interactions between multiple factors may determine injury potential [24, 100]. Only 11% of the netball studies used multivariate statistics to assess the impact of a range of risk factors on injury, and even these approaches are suggested to be insufficient to identify the complex interactions between multiple risk factors [100].

The mechanisms of injury, or inciting event leading to an injury, has been identified in a number of netball injury studies using a variety of methods. Some studies report the mode of onset as acute or overuse and/or classify the injury mechanism as contact/non-contact. A greater number of studies (45%) describe the injury inciting event, typically through athlete self-report or medical staff report, using pre-determined categories to guide the responses. This approach has provided some valuable information, but it provides only a simplistic description of the injury event and is often limited in accuracy, as it relies on biased retrospective recall [101]. Thus, the understanding of injury inciting events in netball requires further investigation. Thus far, only two studies have conducted a more comprehensive assessment of netball injuries using video-based methods to accurately describe the inciting event. Stuelcken et al. [82] and Belcher et al. [88] assessed the mechanisms of ACL injuries, providing a full description of the playing situation, movement patterns and player behaviour at the time of injury. However, no research to-date has developed video-based methods to assess a wider range of injuries and their causes in netball.

To better understand the aetiology and mechanisms of injury in the second step of the sequence of injury prevention [20, 21], aetiology research should employ a multifactorial approach. This should assess the complex interaction between multiple intrinsic and extrinsic factors, workload and the injury inciting event [24, 102]. Hence, studies need to make use of a dynamic model which describes the interaction between as many risk factors as possible, appropriate workload measures and the events leading to the injury. The multifactorial model additionally needs to account for the dynamic, recursive nature of sports injury. Such models include Windt & Gabbett’s [102] workload-injury aetiology model, developed from the original multifactorial models of Meeuwisse and colleagues [103, 104]. Accurate assessment of netball injury mechanisms, to inform the injury model, require a consistent approach. The development of video-based methods that fully describe the playing situation, player/opponent behaviour and accurately assess the biomechanics of injury are necessary to provide a complete assessment of the injury inciting event. Combining these video methods, where possible, with athlete and medical staff descriptions is recommended to provide a more comprehensive understanding of injury causality [23, 101]. To facilitate clear comparisons between studies, the definitions and terminology recommended in the recent consensus on netball video analysis framework [105] should also be adopted.

Finally, to analyse the non-linear interactions between these injury determinants a complex systems approach has been suggested by Bittencourt et al. [100] to be a more appropriate method of assessing sport injuries. The method identifies a risk profile from the interactions between the “web” of injury determinants. Appropriate statistical methods are necessary to identify injury predictions rather than relationships. These methods include recursive partitioning-based methods e.g. classification and regressions trees (CART) and random forests, or machine/statistical learning methods [100]. Figure 3 summarises the recommendations for netball injury aetiology and mechanism research methodologies. Future research should address these methodological concerns to provide an accurate netball injury evidence base which is critical to inform the development of targeted injury prevention strategies. This study provides a comprehensive summary of the research methodologies describing the extent of the injury problem and aetiology and mechanisms of injuries in netball. However, it is possible the search may not have identified all studies in the area.

## Conclusion

This scoping review reveals a lack of systematic and ongoing injury surveillance systems in the netball injury research describing the injury problem. Studies exhibit considerable heterogeneity in methodologies, including study designs, injury definitions, data collection methods and injury reporting practices. Inconsistent methods of reporting injury rates and classification of study populations further limit the quality of evidence across different age groups and level of play. Research assessing injury aetiology often focuses on a limited number of risk factors, using reductionist approaches, while studies assessing injury mechanisms use simplistic descriptions, based on unreliable retrospective recall. Therefore, additional research is needed to comprehensively assess the netball injury problem, its causes, and mechanisms within the modern game, considering a broader spectrum of playing styles.

Accurately identifying key injury issues in netball, requires reliable and consistent injury surveillance systems across settings. The IOC consensus statement guidelines are recommended for the accurate collection of injury data, providing clear definitions, collection methods and reporting protocols. To understand the causes of netball injuries, a multifactorial approach is essential to assess the complex interaction between multiple intrinsic and extrinsic factors, player load and the injury inciting event. Detailed assessment of the inciting event should encompass the playing situation, player/opponent behaviour, and joint and whole-body biomechanics utilising video analysis and medical staff descriptions.

### Supplementary Information

Additional file 1: Preferred Reporting Items for Systematic reviews and Meta-Analyses extension for Scoping Reviews (PRISMA-ScR) Checklist

Additional file 2: Table 1. Frequency of Netball injury studies by study design and year of publication. Table 2. Frequency of intrinsic and extrinsic risk factors by study design. Fig. 1. Frequency of Netball Injury studies by study design. Fig. 2. Frequency of Netball injury studies by study design and country of origin. Fig. 3. Frequency of Netball injury studies by study design and body region. Fig. 4. Frequency of Netball injury studies by study design and data collection method

## Data Availability

All data generated or analysed during this study are included in this published article and its supplementary information files.

## Abbreviations

CART: Classification and regressions trees
IOC: International Olympic Committee
OSIICS: Orchard Sports Injury Classification System
PRISMA-ScR: Preferred Reporting Items for Systematic reviews and Meta-analyses extension for Scoping Reviews
ROM: Range of Motion
TRIPP: Translating Research into Injury Prevention Practice
