# Supplementary material for: The netball injury evidence base: a scoping review of methodologies and recommendations for future approaches
Source: Syst Rev. 2024 Aug 1;13:203. doi: 10.1186/s13643-024-02629-7 (PMC11295446; doi:10.1186/s13643-024-02629-7)
Supplement: Supplementary file 2 — Additional file 2: Table 1. Frequency of Netball injury studies by study design and year of publication. Table 2. Frequency of intrinsic and extrinsic risk factors by study design. Fig. 1. Frequency of Netball Injury studies by study design. Fig. 2. Frequency of Netball injury studies by study design and country of origin. Fig. 3. Frequency of Netball injury studies by study design and body region. Fig. 4. Frequency of Netball injury studies by study design and data collection method [file 13643_2024_2629_MOESM2_ESM.docx]

Fig S1


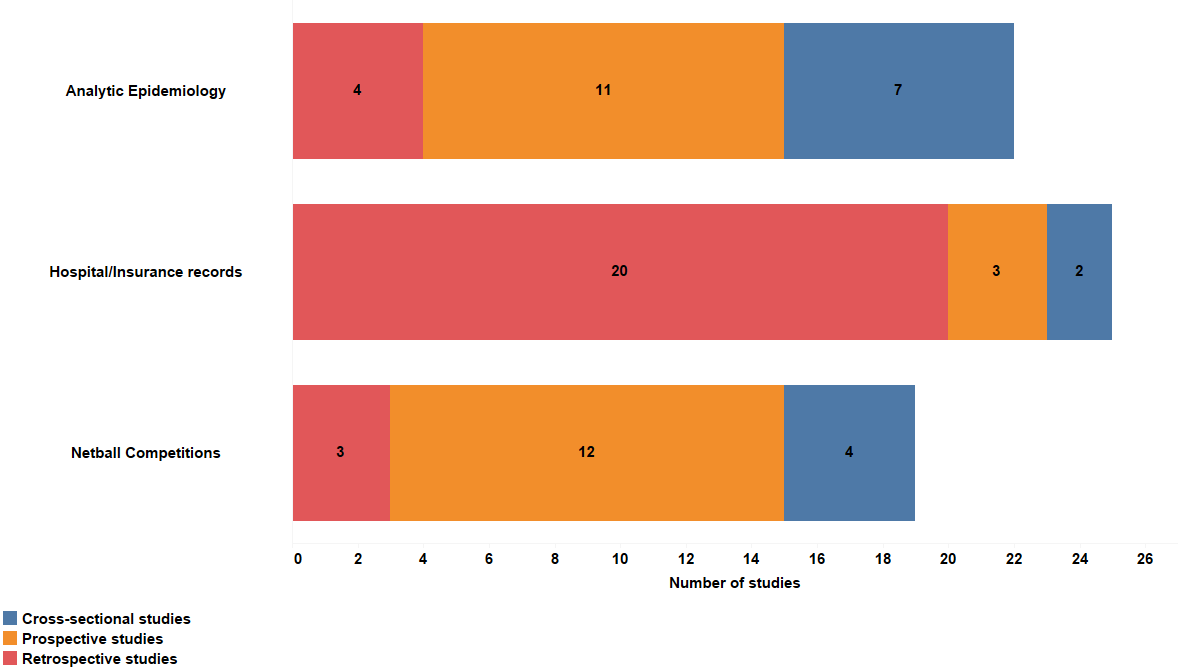


Fig S2


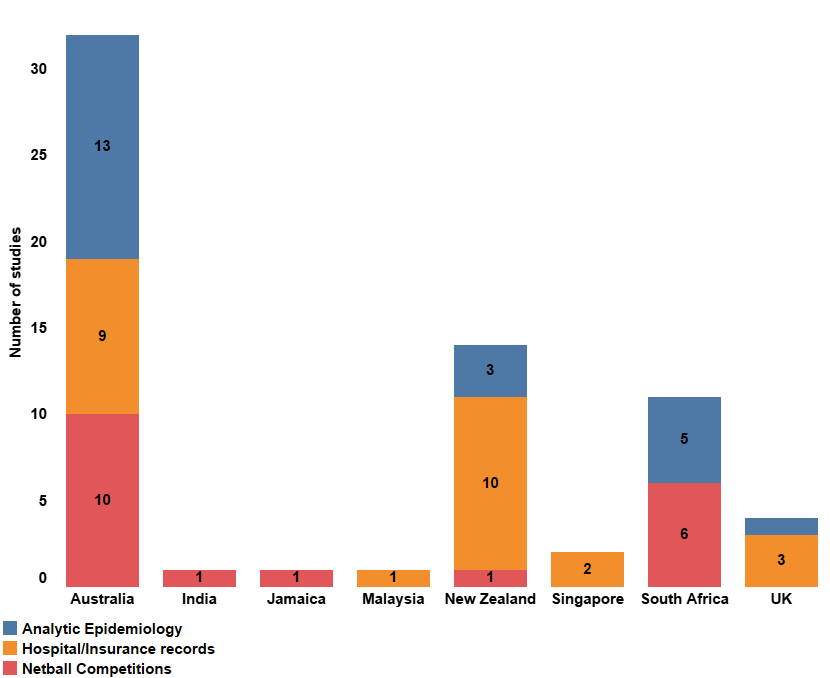


Fig S3


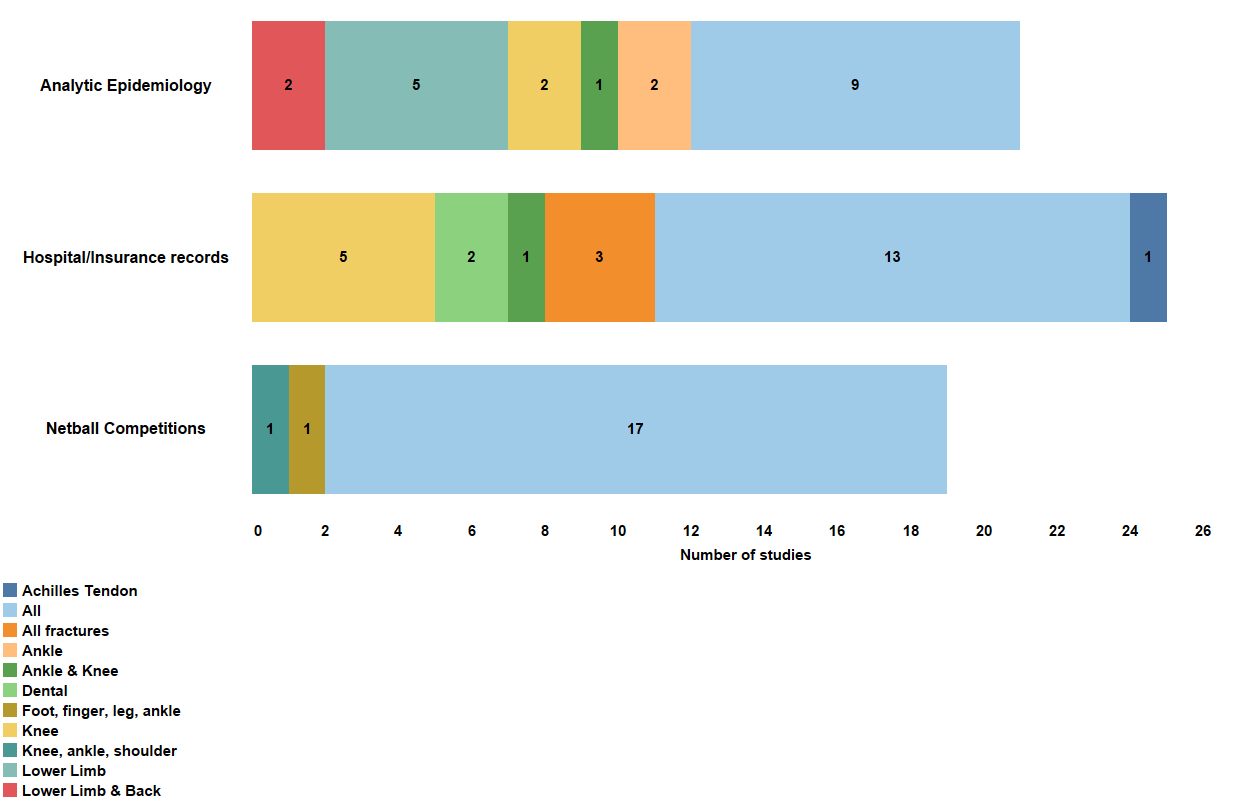


Fig S4


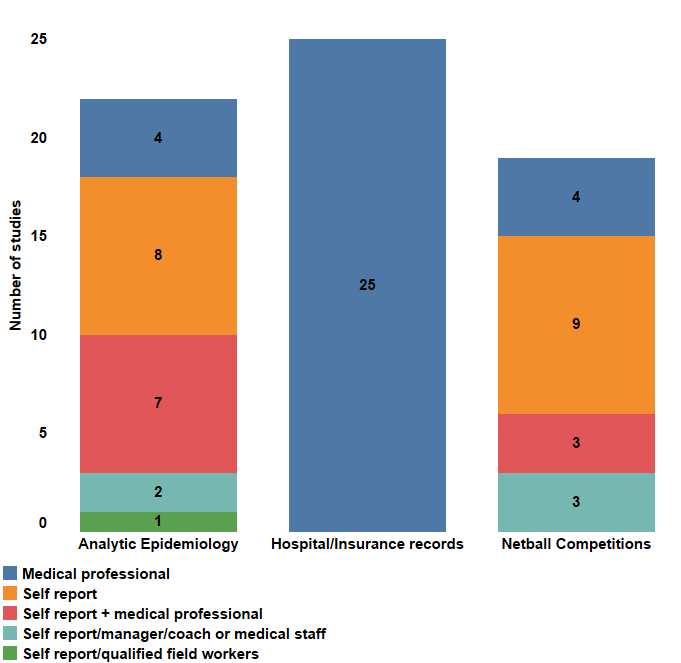


**Table 1:** Frequency of Netball injury studies by study design and year of publication

| Study Design | Pre 1998 | | 1998–2007 | | 2008–2018 | | 2019–May 2024 | |
| --- | --- | --- | --- | --- | --- | --- | --- | --- |
|  | n | % | n | % | n | % | n | % |
| **Descriptive Epidemiology**  Hospital/clinic records/  insurance databases  Netball  competitions | **6**  3  3 | **14**  7  7 | **12**  7  5 | **27**  16  11 | **12**  7  5 | **27**  16  11 | **14**  8  6 | **32**  18  14 |
| **Analytic Epidemiology** | **4** | **19** | **2** | **10** | **8** | **38** | **7** | **33** |

^n = number of studies, % = percentage of studies^

**Table 2:** Frequency of intrinsic and extrinsic risk factors by study design

| **Risk Factors** | **Descriptive**  **Epidemiology** | | **Analytic Epidemiology** | **Total** |
| --- | --- | --- | --- | --- |
|  | Hospital/clinic records  insurance databases | Netball Competitions |  |  |
|  | n | n | n | **n %** |
| **Intrinsic Factors**  Age  Gender  Ethnicity  Position  Previous injury  Injured side  Injury symptoms  Body composition factors:  Height  Mass  BMI  Body fat percentage  Anatomical/biomechanical  factors:  Limb dominance  Limb asymmetry  Foot types  Podiatric measurements  Hypermobility  Weak joints  Ankle joint laxity  Ankle instability  Ankle/dorsiflexion ROM  Ankle isokinetic strength  Lower limb muscle activity  Frontal plane knee angle  Postural stability/balance  Lower body stiffness  Symmetry, dynamic mobility  & local stability  Quadricep/Hamstring ratio  Movement competency  Physiological Factors:  Somatotype  Muscular power  Anaerobic fitness  Aerobic fitness  Agility  Lower back flexibility  Upper & lower body & core  strength  Strength endurance  Speed  **Extrinsic Factors**  Cost of injury  Place of injury  Playing with injury  Court location  No. matches played  Games per week  Playing experience  Level of competition  Footwear  Tape/Braces  Playing surface type  Playing surface condition  Weather conditions  Warm-up/cool-down  Time factors:  Time across years  Time in season  Time following injury  Match half  Match quarter  Time in Quarter  Time playing other sports  Training factors:  Training in previous year  Pre-season training  Weekly training  Training time/volume  Training modality/type  Training load  Prior training  Prior training duration  Training Preparedness  Treatment factors:  Time-loss  Initial treatment required  Further treatment required  Referral type  Physiotherapist referral  Hospital referral  Surgery  Time to surgery  Return to play  Others:  Number of coaches  Players open to new ideas | 12  8  2  1  0  1  0  0  0  0  0  0  0  0  0  0  0  0  0  0  0  0  0  0  0  0  0  0  0  0  0  0  0  0  0  0  0  0  3  1  0  1  0  0  0  0  0  0  1  1  1  1  2  0  0  1  1  0  0  0  0  0  0  0  0  1  1  0  0  0  0  0  1  0  2  1  1  0  0 | 10  2  1  8  3  1  1  1  1  1  0  0  0  0  0  0  0  0  0  0  0  0  0  0  0  0    0  0  0  0  0  0  0  0  0  0  0  0  0  0  1  1  1  0  3  4  3  2  1  0  1  4  1  3  0  0  6  1  0  0  3  8  6  2  0  0  0  0  2  7  2  0  1  1  0  0  0  1  0 | 10  0  1  6  10  4  0  8  8  4  3  2  1  2  1  2  1  1  1  3  1  1  1  5  1  1  1  1  2  5  1  1  2  1  1  1  1  1  0  0  0  0  1  1  2  7  1  3  1  0  0  2  0  3  1  0  4  1  2  1  1  1  2  1  1  0  0  1  2  2  0  1  0  0  0  0  0  0  1 | **32 49**  **10 15**  **4 6**  **15 23**  **13 20**  **6 9**  **1 2**  **9 14**  **9 14**  **5 8**  **3 5**  **2 3**  **1 2**  **2 3**  **1 2**  **2 3**  **1 2**  **1 2**  **1 2**  **3 5**  **1 2**  **1 2**  **1 2**  **5 8**  **1 2**  **1 2**  **1 2**  **1 2**  **2 3**  **5 8**  **1 2**  **1 2**  **1 2**  **2 3**  **1 2**  **1 2**  **1 2**  **1 2**  **3 5**  **1 2**  **1 2**  **2 3**  **2 3**  **1 2**  **5 8**  **11 17**  **4 6**  **5 8**  **3 5**  **1 2**  **2 3**  **7 11**  **3 5**  **6 9**  **1 2**  **1 2**  **11 17**  **2 3**  **2 3**  **1 2**  **4 6**  **9 14**  **8 12**  **3 5**  **1 2**  **1 2**  **1 2**  **1 2**  **4 6**  **9 14**  **2 3**  **1 2**  **2 3**  **1 2**  **2 3**  **1 2**  **1 2**  **1 2**  **1 2** |

^n = number of studies, % = percentage of studies^
